# α‐ZnO Manipulated Growth of Ag Gird on AgNWs Enables High Conductive Flexible Electrode for Large‐Area Monolithic Organic Photovoltaics

**DOI:** 10.1002/advs.202410931

**Published:** 2025-01-22

**Authors:** Zhuo Chen, Tianyu Liu, Irfan Ismail, Fan Qian, Lianping Zhang, Shutao Yang, Xiaoke Zhang, Lingpeng Yan, Yunfei Han, Qun Luo, Yongzhen Yang, Chang‐Qi Ma

**Affiliations:** ^1^ College of Materials Science and Engineering Taiyuan University of Technology Taiyuan 030024 P. R. China; ^2^ i‐Lab & Printable Electronics Research Center Suzhou Institute of Nano‐Tech and Nano‐Bionics Chinese Academy of Sciences(CAS) Suzhou 215123 P. R. China; ^3^ School of Nano‐Tech and Nano‐Bionics University of Science and Technology of China Hefei 230027 P. R. China

**Keywords:** Ag grids, organic solar cells, silver nanowires, vacuum evaporation

## Abstract

The conductivity of AgNWs electrodes can be enhanced by incorporating Ag grids, thereby facilitating the development of large‐area flexible organic solar cells (FOSCs). Ag grids from vacuum evaporation offer the advantages of simple film formation, adjustable thickness, and unique structure. However, the complex 3D multi‐component structure of AgNWs electrodes will exacerbate the aggregation of large Ag particles, causing the device short circuits. To address this issue, the relationship between the surface energy of modification layers and the morphology and conductivity of ultrathin Ag on AgNWs is studied. The amorphous ZnO (α‐ZnO) layer promotes Ag growth from Volmer–Weber (VW) to Frank–Van der Merwe (FM), reducing particle aggregation. The 1 µm thick PET/AgNWs/Ag grid electrode with α‐ZnO exhibited low contact resistance and high conductivity. As a result, 1 cm^2^ FOSCs with Ag grids achieve a power conversion efficiency (PCE) of 16.01%. As the area increased to 4 and 9 cm^2^, the performance of the monolithic FOSCs is 14.70% and 12.69%, showing less efficiency loss during upscaling. The 8 and 16 cm^2^ modules constructed by series and parallel connection of the monolithic devices yield PCEs of 14.47% and 12.92%, respectively. This study offers valuable insights into constructing Ag grids on AgNWs electrodes for highly efficient large‐area FOSCs.

## Graphical abstract

In this work, composite flexible electrodes of AgNWs/Ag grid are developed for large‐area monolithic flexible organic solar cells (FOSCs). The growth of the Ag on the AgNWs electrodes is successfully transferred from Volmer–Weber (VW) to Frank‐Van der Merwe (FM) by modifying the AgNWs with α‐ZnO. With the reduced aggregation of large Ag particles, the electrode performance and device success rate are improved.

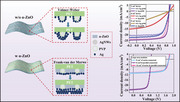

## 1 Introduction

Organic solar cells (OSCs) are attracting significant interest for their advantages of being light, flexible, semi‐transparent, and Roll‐to‐Roll (R2R) fabrication compatible.^[^ ^1^, ^2^, ^3^, ^4^ ^]^ Moreover, the development of non‐fullerene acceptors has led to a significant increase in the power conversion efficiency (PCE) of single‐junction OSCs, with the newest PCE exceeding 20.2%.^[^ ^5^ ^]^ Flexible organic solar cells (FOSCs) have great potential for applications in wearable devices, the Internet of Things, building‐integrated photovoltaics,^[^ ^6^, ^7^, ^8^ ^]^ etc. Currently, the highest PCE of the small‐area (<1 cm^2^) FOSCs has reached 18.5%,^[^ ^9^ ^]^ but the PCE of monolithic devices with an area >4 cm^2^ FOSCs was only 13.08%,^[^ ^10^ ^]^ highlighting a substantial performance gap between large‐area and smaller‐area FOSCs. As the area of the FOSCs increases, a sharp decline in PCE occurs, which is primarily due to the increased conductivity loss.^[^ ^11^ ^]^ Therefore, improving the performance and reproducibility of large‐area FOSCs is crucial for the commercialization of FOSCs.^[^ ^12^, ^13^, ^14^ ^]^

Among the various flexible transparent electrodes (FTEs), AgNWs electrodes are mostly used because of their excellent conductivity, high optical transparency, and superior bending resistance.^[^ ^15^ ^]^ However, the occurrence of uneven and rough junctions, characterized by high contact resistance, adversely affects their overall conductivity.^[^ ^16^, ^17^, ^18^ ^]^ Conventional studies have attempted to improve AgNWs junction contacts and reduce resistance utilizing various welding techniques, including chemical welding,^[^ ^19^ ^]^ plasma welding,^[^ ^20^ ^]^ joule heating,^[^ ^21^, ^22^ ^]^ laser sintering,^[^ ^23^ ^]^ and halide treatment.^[^ ^24^ ^]^ However, these methods have limitations in substantially lowering the resistance of AgNWs electrodes. The introduction of metal grids into the electrode is another good strategy to solve this problem, which allows current collection more effectively. For instance, Wang et al.^[^ ^12^ ^]^ developed the PET/Ag‐grid electrodes as the replacement of traditional PET/ITO electrodes, which reduced charge loss and maintained a PCE of 12.16% for large‐area FOSCs (1 cm^2^) comparable to small‐area rigid devices (0.04 cm^2^). Similarly, Qin et al.^[^ ^25^ ^]^ fabricated poly(ethylene terephthalate)/Ag grid/AgNWs: zinc‐chelated polyethyleneimine (PEI‐Zn) composite electrodes, achieving a PCE of 13.2% for large‐area (54 cm^2^) FOSCs due to the excellent electrical performance of the grid electrodes. Han et al.^[^ ^26^ ^]^ deposited amorphous ITO (α‐ITO) on PET/Ag/Cu‐grid electrodes to prepare PET/Ag/Cu‐grid/α‐ITO electrodes with excellent electrical performance, successfully maintaining high PCE as the area increased. The metal grid electrode demonstrated a sheet resistance (*R*_S_) as low as 1 Ω/sq with a similar approach.^[^ ^27^, ^28^ ^]^ Thus, constructing metal grids on AgNWs electrodes can enhance the electrical performance of AgNWs, improve the current collection, and consequently reduce the PCE loss of large‐area FOSCs.^[^ ^29^ ^]^

Several techniques have been developed for fabricating metal grids, including vacuum evaporation,^[^ ^30^ ^]^ RF magnetron sputtering,^[^ ^31^ ^]^ microcontact printing,^[^ ^32^ ^]^ photolithographic masking,^[^ ^33^ ^]^ and inkjet printing.^[^ ^34^ ^]^ Among these, vacuum evaporation offers significant advantages, such as a simple and well‐controllable film formation system, and high film purity.^[^ ^35^ ^]^ For the evaporation route, the nucleation and growth of the metal films could be explained by one of the three classical mechanisms: Volmer–Weber (VW), Frank‐Van der Merwe (FM), or Stranski‐Krastanow (SK) mechanism.^[^ ^36^ ^]^ The VW mechanism is characterized by the formation of island‐like structures, resulting in less uniform films and affecting their overall performance. The FM mechanism, on the other hand, is favorable for the growth of films with excellent flatness on substrates, thereby facilitating the formation of high‐quality films. The SK mechanism initially involves the formation of a smooth monolayer film (FM), followed by island‐like growth on the film surface (VW), which might introduce defects during the island growth component. Depending on the relative strength of interactions between metal atoms and the substrate. The wettability of the substrate plays a critical role in determining the continuity of the metal electrode and the formation of island structures. A high substrate surface energy will result in FM growth of the Ag film, while a low substrate surface energy will lead to VW growth of the Ag film.^[^ ^37^ ^]^ The AgNWs network electrode film, composed of AgNWs, PVP, and PET substrates, forms a heterogeneous, rough, and complex 3D system. On the top of AgNWs electrodes, Ag particles are more likely to exhibit VW growth, resulting in island‐like structures that can lead to issues such as punctures or short circuits in FOSCs.^[^ ^38^ ^]^ The complex surface state of the AgNWs electrodes results in increased contact resistance, impeding current flow from AgNWs to Ag grids and thereby affecting the overall conductivity of AgNWs/Ag‐grid electrodes. Therefore, it is important to investigate the growth mechanisms of Ag grids on AgNWs electrodes and suppress VW mode growth from good electrical contact between the Ag grid and AgNWs.

In this work, a composite electrode of PET/AgNWs/α‐ZnO/Ag grid was developed for the fabrication of large‐area monolithic cells. The Ag grid was deposited onto the AgNWs electrodes via vacuum thermal evaporation for the conductivity improvement of AgNWs electrodes. To overcome the problem of Ag grid growth through the VW route on the AgNWs due to the complex surface state and a 3D multi‐component structure of AgNWs electrodes, the α‐ZnO modification layer is pre‐deposited on the AgNWs electrodes as a wetting layer. The introduction of α‐ZnO effectively changed the surface state of the AgNWs film, leading to the forming of a smooth Ag grid with minimal particle aggregation and reduced contact resistance on AgNWs. Based on the optimized contact of the Ag grid and AgNWs through α‐ZnO wetting, the relationships between the geometric structure of the grid (grid number, space, and width) efficiency loss were further investigated, and an optimized structure was developed on a 4 cm^2^ electrode, with a grid width of 0.25 mm, space of 4 mm. As a result, monolithic FOSCs with areas of 1, 4, and 9 cm^2^ achieved a PCE of 16.01%, 14.70%, and 12.69%, respectively. The PCE of the 8 and 16 cm^2^ large‐area module from series and parallel connection of the 4 cm^2^ monolithic cells showed efficiency of 14.47%, and 12.92%, respectively. This work provides an effective method for realizing large‐area monolithic FOSCs, and it is also feasible to achieve large‐area modules based on large‐area monolithic FOSCs.

## 2 Results and Discussion

### 2.1 Growth Mechanism of Ag Films and Construction of Ag Grids on the AgNWs

The PET/AgNWs electrodes with a sheet resistance of 10 Ω/sq were fabricated by gravure printing according to the previous works.^[^ ^39^ ^]^ To improve the conductivity of AgNWs electrodes, Ag grids were deposited onto AgNWs, which were then utilized as the composite transparent electrode for the fabrication of large‐area FOSCs. However, the FOSCs based on the PET/AgNWs/Ag‐grid electrodes exhibited severe leakage and even short‐circuit. As illustrated in Figure S1a,b (Supporting Information), the phenomenon of burning and perforation occurred in the grid areas during testing. It can also be seen in Figure S2a,b (Supporting Information) that even with a thick ETL covering, large particle aggregation was still visible on the Ag grids. This issue primarily results from severe aggregation of larger Ag particles on the grid due to the larger surface energy difference of AgNWs composed of AgNWs, PVP, and PET substrate. (Which will be demonstrated later).

Extensive studies have shown that depositing silver on metal oxides can lead to the formation of denser and smoother silver films.^[^ ^40^, ^41^ ^]^ Specifically, It has been reported that α‐ZnO can modify AgNWs, fill in the gaps, reduce roughness, and improve electrode conductivity. Additionally, the inclusion of dual ETLs in inverted devices also contributes to enhanced device performance.^[^ ^10^, ^42^ ^]^ Hence, to solve the problem of the complex surface state of the AgNWs electrodes and suppress the VW mode growth of Ag films on AgNWs electrodes, α‐ZnO metal oxide electron transporting layer (ETL) material was chosen for modifying and growing Ag films.^[^ ^10^ ^]^ Before the fabrication of the composite electrode, the influence of α‐ZnO on the transmittance and root mean square (RMS) roughness should be excluded. Thus, we first study the transmittance spectra and the roughness of the films. Figure S3 (Supporting Information) showed that the transmittance at 550 nm (T_550_) of initial AgNWs was almost 92.9%, and T_550_ of AgNWs/α‐ZnO remained at 92.1%. The root mean square (RMS) roughness of PET/AgNWs electrodes after α‐ZnO modification decreased from 22.4 to 10.7 nm (Figure S4, Supporting Information). These results indicate that the amorphous oxide has a relatively slight effect on the transmittance of the silver electrode, but it can effectively reduce the surface roughness of the electrode.

**Figure 1:**
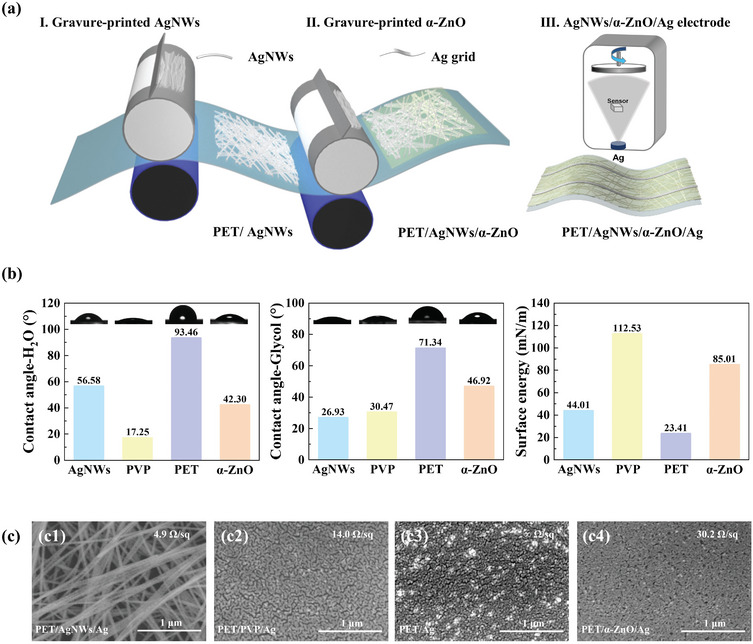
a) The preparation process schematic of the PET/AgNWs/α‐ZnO/Ag‐grid composite electrodes. b) Contact angle images on the top of water and glycol and surface energy of different films. c) SEM morphology and sheet resistance of 10 nm Ag films growing on the PET/AgNWs, PET/PVP, PET, and PET/α‐ZnO films.

With the reduced roughness in hand, we can speculate better electrical properties, thus the composite electrodes were prepared through a process as depicted in **Figure** **1**d. Initially, the PET/AgNWs films and PET/AgNWs/α‐ZnO films were fabricated using roll‐to‐roll gravure printing technology. Next, the PET/AgNWs/Ag grids composite electrodes were created by depositing patterned Ag onto the PET/AgNWs/α‐ZnO films through vacuum thermal evaporation.

The nucleation and growth mechanism of Ag was different due to the difference in surface energy of AgNWS, PVP, PET, and α‐ZnO. To investigate the growth mechanism of Ag films on AgNWs electrodes, ultra‐thin Ag films with a thickness of 10 nm were deposited on AgNWs, PVP, PET, and α‐ZnO surfaces. Contact angle images on the top of water and glycol and surface energy of different films were shown in Figure 1b. The surface energies of PVP, pure AgNWs, PET, and α‐ZnO films were 112.53, 44.01, 23.41, and 85.01 mN/m, respectively. At the same time, the SEM images and R_S_ of 10 nm Ag films growing onto the PET/pure AgNWs, PET/PVP, PET, and PET/α‐ZnO films are shown in Figure 1c. Notably, The *R*_S_ of PET/AgNWs electrodes decreased from the original 10 to 5.0 Ω/sq after depositing 10 nm Ag films, which illustrated the conductivity of AgNWs electrodes could be improved by constructing the AgNWs/Ag composite electrodes. The PET/PVP/Ag films showed a smooth and dense morphology with an R_S_ of 14.0 Ω/sq. The PET/Ag films, however, displayed a discontinuous and aggregated morphology with an R_S_ of ∞ Ω/sq. The PET/α‐ZnO/Ag films showed a continuous yet slightly aggregated morphology with an R_S_ of 30.2 Ω/sq. During the growth process of the silver film electrodes, it was observed that silver atoms preferentially deposited onto films with larger surface energy, as noted in prior reports.^[^ ^43^ ^]^ Hence, Ag atoms were preferentially deposited onto PVP, followed by α‐ZnO, AgNWs, and finally onto PET. The larger variation in wettability without α‐ZnO modification leads to uneven Ag particle growth and larger Ag particle aggregation. Although the morphology and conductivity of ultrathin Ag films can be effectively improved via depositing additional PVP modification layer onto the AgNWs surface. The PVP modification layer would also isolate charge collection between the ETL and AgNWs due to the non‐conductive characteristics of PVP materials, which would cause a dramatic drop in FOSCs performance.^[^ ^44^ ^]^ However, the blank PET and PVP‐wrapped AgNWs in AgNWs electrodes could be covered after the α‐ZnO modification. As a result, the Ag films would tend to grow on the α‐ZnO layer with higher surface energy, which might smooth the surface and regulate the growth process of the Ag grid.

**Figure 2:**
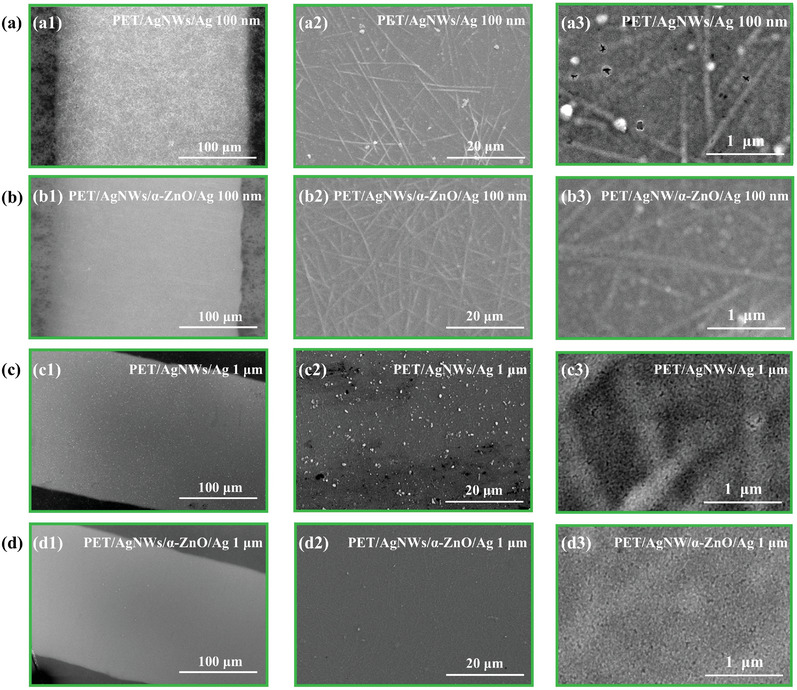
SEM morphology images of Ag grids growth on the different films: a) PET/AgNWs and b) PET/AgNWs/α‐ZnO films with 100 nm thickness varied different magnifications, c) PET/AgNWs and d) PET/AgNWs/α‐ZnO with 1 µm thickness varied different magnifications.

To further explore the effects of α‐ZnO modification on the growth of the thicker Ag grid, SEM images of the PET/AgNWs/Ag‐grid and PET/AgNWs/α‐ZnO/Ag‐grid electrodes with a thickness of 100 nm were measured and shown in **Figure** **2**a,b. As displayed in Figure 2a1–2a3, the surface of the Ag grid in the PET/AgNWs film is rough and sparse, and some regions are rather loose and contain nanoparticle clusters. In contrast, the Ag grids in the PET/AgNWs/α‐ZnO films exhibited a notably smooth and dense surface (as shown in Figure 2b). Subsequently, the Ag grid with a thickness of 1 µm growing on the PET/AgWNs with and without α‐ZnO modification was fabricated and SEM images were investigated. As shown in Figure 2c,d, for the PET/AgNWs/Ag grid electrodes, uneven Ag particle aggregation was evident at a 100 µm scale of SEM, and AgNWs and non‐dense aggregated particles were visible at 20 µm and 400 nm scales. In contrast, a much smoother and dense Ag grid grew on the PET/AgNWs/α‐ZnO films as shown in Figure 2d.

For the fabrication of FOSCs, the top electrode might connect with the Ag grid due to the high step height of the roughness and high step of AgNWs, which would cause a short circuit of the devices. Hence, a LiF layer as an insulation layer is deposited on the top of the Ag grid to cover the Ag grid well through vacuum thermal evaporation. As shown in Figure S6a–c (Supporting Information), significant particle aggregation, and poor LiF coverage were exhibited without α‐ZnO modification, which would increase the risk of leakage or short‐circuit of cells. Conversely, Figure S6d–f (Supporting Information) illustrate that uniform LiF film without particle aggregation was effectively shown with α‐ZnO modification. Figure S7 (Supporting Information) shows the microscope image of short‐circuit and normal FOSC. The FOSC based on the PET/AgNWs/Ag/LiF electrode without α‐ZnO modification showed ablated, volcanic‐like holes, which is due to the Ag grids not being covered well by LiF due to larger Ag particle aggregation.

**Figure 3:**
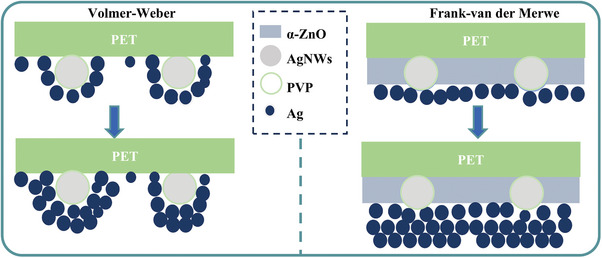
Schematic diagram of Ag grid growth mechanism on the AgNWs electrode without and with the α‐ZnO modification.

Based on the above research results, schematic diagrams of Ag grid growth on the surfaces of PET/AgNWs without and with α‐ZnO modification layer could be described in **Figure** **3**. On the top of AgNWs electrode, the typical network structure, uneven surface, and multiple component materials led to excessive roughness and large surface energy differences and formed heterogeneous surface of physical and chemical. In such a complex surface state, the Ag nanoparticles tend to grow through the VM growth model. As a result, the thick Ag grids growing on the AgNWs surface exacerbated excessive particle aggregation. After the introduction of the α‐ZnO modification layer, the void areas of AgNWs were filled, blank PET areas were covered, the uneven surface of Ag nanowires was flattened, and the surface energy was more homogenous. Additionally, the α‐ZnO films had abundant amine groups, which can provide the nucleation sites for Ag atoms during the vacuum evaporation as reported by literature.^[^ ^45^ ^]^ The physical and chemical heterogeneous properties of AgNWs electrodes were alleviated. The Ag grid growth model was changed from VW to FM due to these improvements. Hence, the Ag grids growing PET/AgNWs/α‐ZnO films showed a uniform and less Ag particle aggregation morphology.

### 2.2 The Universality of Amorphous Oxide Assistant Ag growth

Besides α‐ZnO, other metal oxide materials including ZnO nanoparticles (ZnO NPs), SnO_2_ nanoparticles (SnO_2_ NPs), NiO nanoparticles (NiO NPs), amorphous SnO_2_ (α‐SnO_2_), and amorphous NiO (α‐NiO) films were also chosen to regulate the growth of the thin Ag films. The SEM images and *R*_S_ of 10 nm Ag films growing onto the different metal oxide films are shown in Figure S5 (Supporting Information). The Ag films growing onto the amorphous modification layer behave in a denser and smoother morphology comparing the Ag films growing onto the nanoparticles which exhibited large particle aggregation and isolation. The *R*_S_ of Ag films growing onto the ZnO NPs, α‐ZnO, SnO_2_ NPs, α‐SnO_2_, NiO NPs, and α‐NiO films were 65.9, 30.2, 50.9, 43.1, 126.3, and 31.9 Ω/sq, respectively. Obviously, the Ag films growing onto the amorphous modification layer had a lower *R*_S_ compared to the Ag films that grow onto the nanoparticle modification layer. Based on these results, it can be demonstrated that the amorphous metal oxides might be more suitable for the growth of Ag films.

### 2.3 The Electrical Properties of the Composite Electrodes

**Figure 4:**
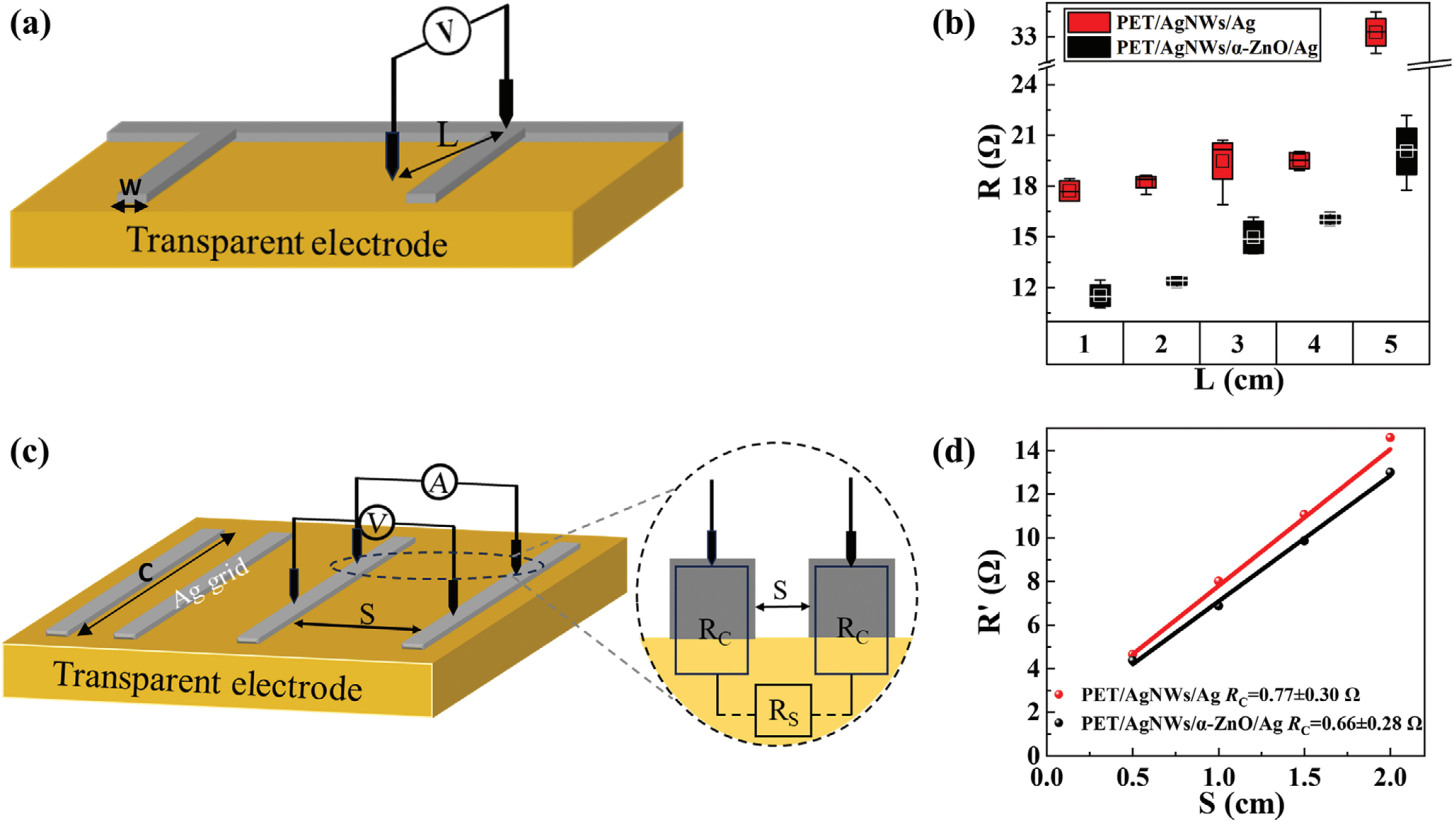
a) Schematic diagram of R as a function of L from the grids on the edge to the internal AgNWs. b) Box plot of R‐value as a function of L. c) Schematic diagram of TLM measurements for the characterization of R_C_ between the AgNWs electrode and the Ag grid. d) The R' value as a function of S for PET/AgNWs/Ag and PET/AgNWs/α‐ZnO/Ag electrode.

The patterned Ag grids were deposited on the top of PET/AgNW electrodes to improve the charge collection, where edge Ag grids served as bus bars and internal Ag grids functioned as finger bars. As shown in **Figure** **4**a, the electrical characteristic of the PET/AgNWs/Ag (Ag grid with a 500 nm thickness) electrodes without and with α‐ZnO modification layer was subsequently investigated by measuring the overall series resistance (R) as a function of length (L) from the grids on the edge to the internal AgNWs. As shown in Figure 4b, R values of distances of 1, 2, 3, 4, and 5 cm were 17.7, 18.24, 19.49, 19.51, and 33.26 Ω for PET/AgNWs/Ag electrode. While for the PET/AgNWs/α‐ZnO/Ag electrode, the R values for distances of 1, 2, 3, 4, and 5 cm were 11.54, 12.38, 14.99, 16.03, and 20.05 Ω, respectively. These results suggested the use of α‐ZnO modification could enhance the electrical characteristics of the composite electrodes and improve the charge transport of monolithic large‐area cells. Additionally, contact resistance (R_C_) between the Ag grids and the PET/AgNWs and PET/AgNWs/α‐ZnO layers was studied using the transfer length method (TLM) measurements.^[^ ^46^ ^]^ The TLM measurement schematic diagram is shown in Figure 4c. As shown in Figure 4b, the total resistance (R') as a function of various grid spaces (S is 0.5, 1, 1.5, and 2 cm) was measured, and fitted using a linear function of $R=\frac{R_{S}}{C}\;S+2R_{C}$. It is observed that when S is zero, R' is twice of R_C_. The R_C_ of PET/AgNWs/Ag and PET/AgNWs/α‐ZnO/Ag grids were 1.53 and 1.33 Ω, respectively. The α‐ZnO modification layer led to smoother and denser Ag grids on the AgNWs, improving contact and reducing contact resistance.

### 2.4 FOSCs Performance Based on PET/AgNWs/α‐ZnO/Ag‐Grid Electrodes

**Figure 5:**
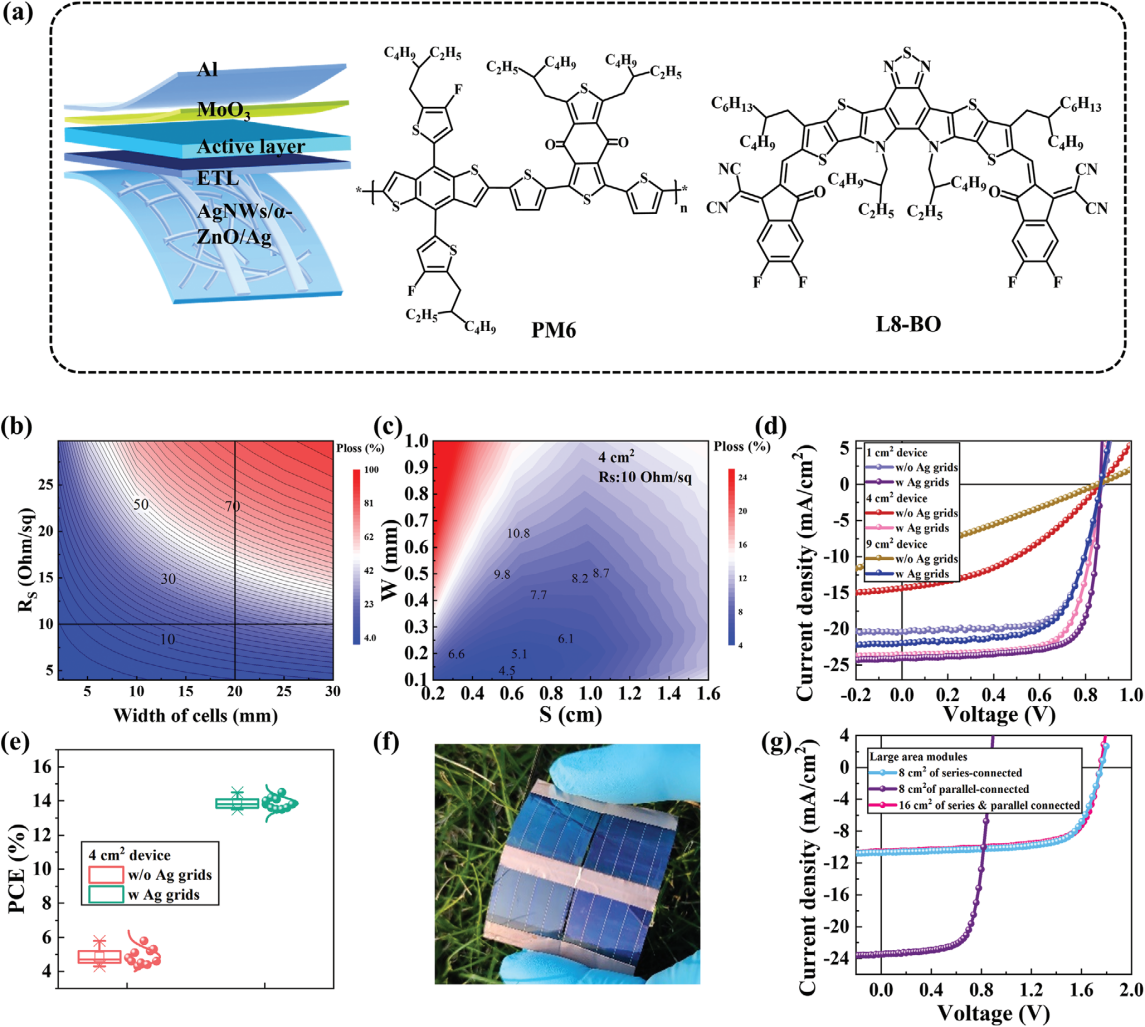
a) The device structure diagram and the molecular structure of the donor PM6 and the acceptor L8‐BO. b) PCE loss as a function of cell width and sheet resistance of FTEs in traditional interconnect contact at the edge. c) Optimization for PCE loss of 4 cm^2^ device as a function of width (W) and space (S) of Ag grids on a conventional electrode with a sheet resistance of 10 Ω/sq. d) The *J–V* characteristics of FOSCs with different areas of monolithic cells without and with Ag grid. e) Statistical PCE distribution of the 4 cm^2^ devices fabricated without and with Ag grids (each data was extracted from 10 cells). f) Photograph of a module composed of four 4 cm^2^ FOSCs. g) The *J–V* characteristics of modules with different areas.

To validate the application potential of the PET/AgNWs/α‐ZnO/Ag‐grid composite electrodes for large‐area FOSCs, flexible devices with a structure of PET/AgNWs/α‐ZnO/Ag‐grid/ETL/PM6:L8‐BO/MoO_3_/Al were fabricated. The device schematic diagram and molecular structures of donor PM6 and acceptor L8‐BO are shown in **Figure** **5**a. Before fabricating the flexible devices, the theoretical Joule heating loss was calculated to see the influence of the Ag grid on efficiency losses. In the case of traditional interconnect contact at the edge in the AgNWs electrode‐based FOSCs, current flows unidirectionally toward the edge. Joule heat loss is significantly large due to excessive lateral current flow from FTEs in this configuration. Particularly, Joule heat loss dramatically increases as the width of cells increases to >1 cm. When Ag finger bars or Ag grids are introduced in the AgNWs electrodes, the Ag grids would act like highways to help charge collection from FTEs, thereby the issues of hindered charge collection due to excessively longer electrode space.

To optimize the Ag grid electrode structure, efficiency loss as a function of width (W) and space (S) of Ag grids was first investigated. Take the device with a width of 2 cm as an example, PCE loss as a function of cell width and *R*_S_ of FTEs in traditional interconnect contact at the edge as shown in Figure 5b. For the FOSCs with a width of 2 cm based on the PET/AgNWs electrode (10 Ω/sq), we found the PCE loss was 22%. Conversely, the PCE loss of the 4 cm^2^ FOSCs with PET/AgNWs/Ag‐grid electrodes could be reduced to 4%. The relationship between efficiency loss and space of the Ag grid at different Ag grid widths is discussed in detail in Figure S8 (Supporting Information). We can see the narrower the Ag grid width is, the smaller the optimized efficiency loss. When the width of the Ag grid is large, the optical loss becomes large. Although narrower grids might further reduce PCE loss, the precision limitations of the mask limit the width of the Ag grid in the vacuum evaporation process. Therefore, the large area monolithic cells based on PET/AgNWs/Ag‐grid electrode were fabricated to achieve minimal PCE loss, where the configuration of Ag grid is 0.25 mm width 4 mm gird space and the optical loss caused by the grids on the monolithic large area FOSCs was only 5%.

To explore the application of PET/AgNWs/α‐ZnO/Ag‐grid electrodes in large‐area monolithic cells, the PET/AgNWs and PET/AgNWs/α‐ZnO/Ag‐grid structures were prepared as FTE for the monolithic FOSCs with different areas. The *J–V* characteristics are shown in Figure 5d and the detailed performance parameters are shown in **Table** **1**. The 1 cm^2^ AgNWs FOSCs without Ag grids exhibited an open‐circuit voltage (*V*_OC_) of 0.879 V, a short‐circuit current density (*J*_SC_) of 20.61 mA cm^−2^, a fill factor (FF) of 67.82%, and a PCE of 12.29%. In contrast, the 1 cm^2^ FOSCs with Ag grids showed a *V*_OC_ of 0.870 V, a *J*_SC_ of 24.24 mA cm^−2^, an FF of 75.93%, and a PCE of 16.01%. The external quantum efficiency (EQE) spectra of the FOSCs without and with grids for an area of 1 cm^2^ were exhibited in Figure S9 (Supporting Information). The integral current of the EQE spectra for the FOSCs without and with grids is 20.88 and 24.25 mA cm^−2^, respectively, which is consistent with the current of the *J‐V* characteristics.

**Table 1:** Device performance of 1, 4, and 9 cm^2^ FOSCs without and with Ag grids. The performance of different area modules composed of 4 cm^2^ FOSCs.

Electrode	Area [cm^2^]	*V*_OC_ [V]	*J*_SC_ [mA cm^−2^]	FF [%]	PCE [%]
PET/AgNWs	1.0	0.879 0.883 ± 0.005	20.61 20.01 ± 0.47	67.82 66.94 ± 1.95	12.29 11.82 ± 0.39
PET/AgNWs/α‐ZnO/Ag		0.870 0.873 ± 0.004	24.24 24.18 ± 0.50	75.93 74.08 ± 2.30	16.01 15.63 ± 0.28
PET/AgNWs	4.0	0.871 0.871 ± 0.002	14.48 14.35 ± 0.46	40.36 40.21 ± 0.32	5.09 4.95 ± 0.56
PET/AgNWs/α‐ZnO/Ag		0.876 0.875 ± 0.004	23.77 23.51 ± 0.51	70.58 70.11 ± 0.66	14.70 14.57 ± 0.71
PET/AgNWs	9.0	0.861 0.861 ± 0.001	9.83 9.80 ± 0.04	27.09 27.06 ± 0.05	2.29 2.28 ± 0.01
PET/AgNWs/α‐ZnO/Ag		0.873 0.868 ± 0.006	22.19 22.25 ± 0.08	65.53 64.29 ± 1.76	12.69 12.42 ± 0.38
PET/AgNWs/α‐ZnO/Ag	8.0^a^ ^)^	0.878 0.877 ± 0.001	23.44 23.27 ± 0.51	70.33 69.73 ± 0.85	14.47 14.22 ± 0.38
	8.0^b^ ^)^	1.757 1.752 ± 0.007	10.70 10.75 ± 0.06	70.07 69.55 ± 0.75	13.18 13.09 ± 0.12
	16.0^c^ ^)^	1.750 1.738 ± 0.008	10.60 10.22 ± 0.25	69.64 69.37 ± 0.18	12.92 12.33 ± 0.39

^a)^ two 4 cm^2^ cells were connected in parallel.

^b)^ two 4 cm^2^ cells were connected in series.

^c)^ four 4 cm^2^ cells were connected in series and parallel hybrid.

In addition, the 4 and 9 cm^2^ monolithic FOSCs were fabricated. The 4 cm^2^ devices without grids exhibited a *V*_OC_ of 0.871 V, a *J*_SC_ of 14.48 mA cm^−2^, an FF of 40.36%, and a PCE of 5.09%. In contrast, the 4 cm^2^ FOSCs with grids showed a *V*_OC_ of 0.876 V, a *J*_SC_ of 23.77 mA cm^−2^, an FF of 70.58%, and a PCE of 14.70%. Similarly, the 9 cm^2^ monolithic cells without Ag grids exhibited a *V*_OC_ of 0.861 V, a *J*_SC_ of 9.83 mA cm^−2^, an FF of 27.09%, and a PCE of 2.29%. By contrast, the 9 cm^2^ monolithic FOSCs with grids showed a *V*_OC_ of 0.873 V, a *J*_SC_ of 22.19 mA cm^−2^, an FF of 65.53%, and a PCE of 12.69%. The devices with grids showed a significant advantage over the devices without grids in the case of 4 and 9 cm^2^ cells. However, with the increase of area from 1 to 9 cm^2^, an increase in charge loss was observed, which might be due to the unfavorable conduction of the vacuum‐evaporated Ag grid.

For further analysis of the device physics, the 4 cm^2^ FOSCs with and without Ag grids were studied. Figure S10a (Supporting Information) shows the optimal photocurrent density (*J*_ph_) versus the effective voltage (*V*_eff_) characteristics of these devices. Here, *J*_ph_ was defined as *J*_L_–*J*_D_ (where *J*_L_ and *J*_D_ were the current densities under illumination and dark conditions, respectively), and *V*_eff_ was defined as *V*_0_–*V* (where *V*_0_ was the voltage at *J*_ph_ = 0 and *V* was the applied voltage). At sufficiently high *V*_eff_, *J*_ph_ reaches saturation without recombination (*J*_ph, sat_). The ratio of *J*_ph_ to *J*_ph, sat_ reflects the probability of exciton dissociation and charge collection. Under short‐circuit conditions, both devices with and without grids exhibited a 97% *J*_ph, sc_/*J*_ph, sat_ ratio, indicating that exciton dissociation efficiency is similar regardless of grid presence. Under maximum output power conditions, the device without grids showed a 47% *J*_ph, max_/*J*_ph, sat_ ratio, while the device with grids exhibited 84% *J*_ph, max_/*J*_ph, sat_, demonstrating that Ag grids significantly enhance charge extraction and collection in large‐area FOSCs. Figure S10b (Supporting Information) presents the Electrochemical Impedance Spectro (EIS) impedance spectra for 4 cm^2^ FOSCs with and without grids. Both spectra show asymmetric semicircles and equivalent circuit fitting was used for analysis. The equivalent circuit model comprised three resistors (R_S_, the series resistance; R_trans_, the transport resistance; and R_rec_, the recombination resistance) and two capacitors (C_trans_ and C_rec_). The fitting parameters of the electrochemical impedance of PET/AgNWs and PET/AgNWs/α‐ZnO/Ag‐grid FOSCs were summarized in Table S1 (Supporting Information). For FOSCs with AgNWs electrodes without grids, the R_S_ was 10.4 Ω, the R_trans_ was 127.7 Ω, and the R_rec_ was 78.7 Ω. In contrast, FOSCs with grids had an R_S_ of 2.9 Ω, an R_trans_ of 42.4 Ω, and a R_rec_ of 2556 Ω. This result indicates that grids significantly reduce the R_S_ of the FOSCs, which would be due to improved conductivity and lower R_trans_. However, the increased charge transport efficiency to the external circuit results in a higher R_rec_. These results indicate that the devices without Ag grids had low *J*_SC_, FF, and PCE due to higher charge transport resistance. In contrast, the devices including grids had improved *J*_SC_, FF, and PCE, which demonstrated that Ag grids effectively reduce charge transport loss.

It is all known that the performance statistics of the devices are important as they demonstrate the reproducibility and reliability of the devices. Statistical PCE distribution of the 4 cm^2^ devices fabricated without and with Ag grids (each data was extracted from 10 cells) was exhibited in Figure 5e. It can be observed that the efficiency of the 4 cm^2^ devices without grids ranges from 4.3% to 5.8%, while the efficiency of the devices with grids ranges from 13.5% to 14.7%. This result means the devices with grids enable higher efficiency and good reproducibility.

Then, the large‐area modules composed of 4 cm^2^ monolithic cells connecting through series, parallel, and series & parallel hybrid were fabricated. The schematic diagram of the module connections is shown in Figure S11 (Supporting Information) and Figure 5f displays a photograph 16 cm^2^ large area module. The *J‐V* characteristics of these modules are shown in Figure 5g and the corresponding device performance is presented in Table 1. The 8 cm^2^ module containing two 4 cm^2^ monolithic cells in parallel connected exhibited a *V*_OC_ of 0.878 V, a *J*_SC_ of 23.44 mA cm^−2^, an FF of 70.33%, and a PCE of 14.47%. The 8 cm^2^ module containing two 4 cm^2^ monolithic cells in series connection presented a *V*_OC_ of 1.757 V, a *J*_SC_ of 10.70 mA cm^−2^, an FF of 70.07%, and a PCE of 13.18%. The 16 cm^2^ containing four 4 cm^2^ monolithic cells in series & parallel hybrid connection showed a *V*_OC_ of 1.750 V, a *J*_SC_ of 10.60 mA cm^−2^, a FF of 69.64%, and a PCE of 12.92%. The performance retention rate from monolithic cells to large area modules is relatively good, which demonstrates the development of monolithic cells is necessary for the realization of the large area FOSCs modules.

## 3 Conclusion

In summary, The α‐ZnO modification layer was selected to modify the AgNWs for the growth of Ag grids. The growth mechanism of Ag deposited by vacuum thermal evaporation was successfully transformed from Volmer‐Weber (VW) to Frank‐Van der Merwe (FM), which inhibited the aggregation of large Ag particles. As a result, PET/AgNWs/α‐ZnO/Ag grid electrodes with low contact resistance and high conductivity were designed and fabricated. Through the optimization of the grid structure, PET/AgNWs/Ag‐grid electrode was designed with grids of 0.25 mm width, and 4 mm space. In contrast to the control device without Ag grids (with a PCE of 12.29%), 1 cm^2^ FOCSs with Ag grids showed a promoted PCE of 16.01%. With area increased to 4.0, and 9.0 cm^2^, the monolithic devices still display a performance of 14.70%, and 12.69%, respectively, showing less efficiency loss during upscaling. Moreover, the large‐area FOSCs modules were fabricated using the 4 cm^2^ monolithic cells through series and parallel connections. 8 and 16 cm^2^ modules show an efficiency of 14.47%, and 12.92%, respectively. The potential applications of grid electrodes in FOSCs are highlighted in this work and are of certain guidance.

## 4 Experimental Section

### Materials

AgNWs ink (10 mg mL^−1^ in H_2_O or IPA, average diameter ≈ 25 nm, length ≈ 25 µm) was procured from Nanchang Hechuang Advanced Materials Co., Ltd. (Nanchang, China). Zinc acetate was supplied by Aladdin Ltd. (poly((4,8‐bis(5‐(2‐ethylhexyl)‐4‐fluoro‐2‐thienyl)benzo[1,2‐b:4,5‐b′]dithiophene‐2,6‐diyl)‐2,5‐thiophenediyl(5,7‐bis(2‐ethylhexyl)‐4,8‐dioxo‐4H,8H‐benzo[1,2‐c:4,5‐c′]dithiophene‐1,3‐diyl)‐2,5‐thiophenediyl)) (PM6) donor and the material (2,2′‐((2Z,2′Z)‐((3,9‐bis(2‐butyloctyl)‐12,13‐bis(2‐ethylhexyl)‐12,13‐dihydro‐[1,2,5]thiadiazolo[3,4‐e]thieno[2″,3″:4′,5′]thieno[2′,3′:4,5]pyrrolo[3,2‐g]thieno[2′,3′:4,5]thieno[3,2‐b]indole‐2,10‐diyl)bis(methaneylylidene))bis(5,6‐difluoro‐3‐oxo‐2,3‐dihydro‐1H‐indene‐2,1‐diylidene))dimalononitrile) (L8‐BO) acceptor were obtained from HePu Optoelectronic Technology Co., Ltd. Surface‐hydrophilic PET was purchased from Toyobo Co., Ltd., Osaka, Japan. Chloroform was sourced from Damas‐Beta, 2‐methoxyethanol was acquired from J&K Scientific, and ethanolamine was purchased from Sigma‐Aldrich. SnCl_4_·5H_2_O was obtained from Shanghai Aladdin Biochemical Technology Co., Ltd. α‐SnO_2_ was purchased from Xian Yuri Solar Co., Ltd. NiO NPs were sourced from Liaoning Youxuan New Energy Technology Co., Ltd. Ni(NO_3_)_2_·6H_2_O was purchased from Sinopharm Chemical Reagent Co., Ltd. Copper foil tape was purchased from Huizhou Kesheng Industrial Co., Ltd. Zn(OAc)_2_·2H_2_O and KOH were purchased from J&K Scientific Ltd.

### Instruments and Measurements

The current density‐voltage (*J–V*) measurements were performed using a Keithley 2400 source meter under a 100 mW cm^−2^ AM 1.5 G simulated solar light source (XES‐40S3) in a nitrogen glove box. Contact angles of various films were measured with the SDC‐350D automatic tilting contact angle goniometer (CN SINDIN Industry). The morphology of the AgNWs flexible electrodes was measured using the S4800 scanning electron microscope (SEM). Microscope images were observed and taken with a self‐developed nano‐technology microregion detection system. Electrochemical impedance spectroscopy (EIS) tests were conducted using the PGSTAT302N (Netherlands Metrohm Autolab B.V.). The sheet resistance (R_S_) of Ag films on different substrates was tested using a four‐probe device (Suzhou Jingge Electronic). The transmittance spectra of the electrodes before and after α‐ZnO modification were measured using a UV/Vis spectrophotometer (PerkinElmer, lambda750). The surface morphology and roughness of the electrodes before and after α‐ZnO modification were examined in tapping mode using an atomic force microscope (Dimension Icon).

### Preparation of Modifier Materials

The α‐ZnO precursor was prepared by dissolving zinc acetate in dimethoxyethanol, with a small amount of ethanolamine added as a stabilizer, followed by overnight stirring.^[^ ^10^ ^]^ For the conventional low‐temperature processed ZnO NPs synthesized through the chemical reaction of zinc salt and hydroxide.^[^ ^47^ ^]^ The preparation of SnO_2_ NPs involved adding 17.5 g of SnCl_4_·5H_2_O to 100 mL of deionized water and stirring for 0.5 hours to create a SnCl_4_ solution. Then, a 5 M KOH solution was added dropwise, and the mixture was stirred for one hour before aging for 2 days. The supernatant was decanted, and the precipitate was washed three times with deionized water to obtain SnO_2_ nanoparticles. For α‐NiO, 2.91 g of Ni(NO_3_)_2_·6H_2_O was dissolved in 10 mL of ethylene glycol and stirred for 12 hours. α‐SnO_2_ and NiO NPs were purchased from commercial sources.

### Preparation of FOSCs

The PET/AgNWs were synthesized following the method reported by Wang et al.^[^ ^48^ ^]^ To prepare the PET/AgNWs/modifier layer films, the PET/AgNWs electrode was first treated in an oxygen plasma chamber for 2 minutes. Modifier materials ink was then gravure printed onto the PET/AgNWs electrodes using a gravure printer at 50 m min^−1^. The obtained PET/AgNWs/ modifier layer films were annealed at 120 °C for 10 minutes. Then, the PET/AgNWs/modifier layer was placed in the vacuum thermal evaporation chamber, where 1 µm thick, 0.25 mm wide Ag grids were deposited using a mask, creating the PET/AgNWs/modifier layer/Ag‐grid composite electrode. Next, 500 nm of LiF was deposited on top of the grids using the same mask. The FOSCs were fabricated with an inverted structure of PET/AgNWs/modifier layer/Ag‐grid/ZnO NPs/Active layer/MoO_3_/Al. Firstly, A 15 mg mL^−1^ ZnO NPs solution was spin‐coated at 1800 r min^−1^ onto the PET/AgNWs/modifier layer/Ag layer and then annealed at 130 °C for 10 minutes. The active layer inks were fabricated by dissolving the 7.5 mg mL^−1^ PM6 donor and 9 mg mL^−1^ L8‐BO acceptor together in chloroform with 50 wt.% DIB as an additive and stirred at 55 °C for 1 h in the glove box. The active layer inks were deposited onto the ZnO films at 2000 r min^−1^ for 30 s and active layer films were thermally annealed at 85 °C for 5 min. After annealing, the samples were transferred to the evaporation chamber. In the presence of a shadow mask,10 nm MoO_3_ and 200 nm Al were deposited on the samples under a vacuum of less than 1 × 10^−4^ Pa.

### Preparation of the Module

Multiple 4 cm^2^ sub‐cells were assembled into a module using copper foil tape. The specific process involved connecting the bottom electrode of the front FOSC to the top electrode of the rear FOSC in series with copper foil tape, while also allowing the FOSCs arranged on the left and right to be connected in parallel. The schematic diagram of the module connections is shown in Figure S11 (Supporting Information), which represents (a) two cells in series, (b) two cells in parallel, and (c) four cells in series & parallel hybrid connected.

## Conflict of Interest

The authors declare no conflict of interest.

## Supporting information



Supporting Information

## Data Availability

The data that support the findings of this study are available in the supplementary material of this article.
