# Supplementary material for: α‐ZnO Manipulated Growth of Ag Gird on AgNWs Enables High Conductive Flexible Electrode for Large‐Area Monolithic Organic Photovoltaics
Source: Adv Sci (Weinh). 2025 Jan 22;12(10):2410931. doi: 10.1002/advs.202410931 (PMC11904985; doi:10.1002/advs.202410931)
Supplement: Supplementary file 1 — Supporting Information [file ADVS-12-2410931-s001.docx]

Supporting information

α-ZnO Manipulated Growth of Ag Gird on AgNWs Enables High Conductive Flexible Electrode for Large-area Monolithic Organic Photovoltaics

Zhuo Chen, Tianyu Liu, Irfan Ismail, Fan Qian, Lianping Zhang, Shutao Yang, Xiaoke Zhang, Lingpeng Yan,^*^ Yunfei Han,^*^ Qun Luo,^*^ Yongzhen Yang, Chang-Qi Ma

Z. Chen, X. Zhang, Assoc. Prof. L. Yan, Prof. Y. Yang

College of Materials Science and Engineering, Taiyuan University of Technology, Taiyuan, 030024 P. R. China.

E-mail: yanlingpeng@tyut.edu.cn

Z. Chen, T. Liu, Dr. I. Ismail, F. Qian, L. Zhang, S. Yang, Dr. Y. Han, Dr. Y. Han, Prof. Q. Luo, Prof. C.-Q. Ma

i-Lab & Printable Electronics Research Center, Suzhou Institute of Nano-Tech and Nano-Bionics, Chinese Academy of Sciences(CAS), Suzhou, 215123, P. R. China.

E-mail: [yfhan2017@sinano.ac.cn](mailto:yfhan2017@sinano.ac.cn); [qluo2011@sinano.ac.cn](mailto:qluo2011@sinano.ac.cn)

T. Liu, F. Qian, Prof. Q. Luo, Prof. C.-Q. Ma

School of Nano-Tech and Nano-Bionics, University of Science and Technology of China, Hefei, 230027, P. R. China.

Figure S1 SEM images of failed FOSCs microspheres: (a) Scale bars: 1 mm. (b) Scale bars: 100 μm.

Figure S2. SEM images of (a) PET/AgNWs/α-ZnO/ZnO NPs, (b) PET/AgNWs/Ag grid/α-ZnO/ZnO NPs films.

Figure S3. Transmittance spectra of AgNWs and AgNWs/α-ZnO.

Figure S4. AFM of (a) PET/AgNWs and (b) PET/AgNWs/α-ZnO films.

Figure S5. SEM images and sheet resistance of 10 nm-thick Ag films grown on different metal oxide films: (a) ZnO NPs, (b)SnO_2_ NPs, (c) NiO NPs, (d) α-ZnO, (e) α-SnO_2_, (f) α-NiO.

Figure S6. SEM images of PET/AgNWs/Ag/LiF and PET/AgNWs/α-ZnO/Ag/LiF films, scale bars: (a,d) 3 µm, (b,e) 100 nm, (c,f) 500 nm.

Figure S7. Microscope images of FOSCs: (a) failed, (b) survived.

Figure S8. The relationship between efficiency loss and space of Ag grid at different Ag grid widths.

Figure S9. The EQE spectra of 1 cm^2^ FOSCs with and without grids.

Figure S10. (a) The photocurrent density (*J*_ph_) versus effective voltage (*V*_eff_) characteristics, and (b) the EIS impedance spectra of 4 cm^2^ flexible devices with and without Ag grids.

Figure S11. The schematic diagram of the module connections: (a) two cells in series, (b) two cells in parallel, and (c) four cells in a mixed configuration.

Table S1. Fitting parameters of the electrochemical impedance of PET/AgNWs and PET/AgNWs/α-ZnO/Ag-grid FOSCs.

| Electrode | *R*  [Ω] | *R_1_*  [Ω] | *R_2_*  [Ω] | *C_1_* | *C_2_* |
| --- | --- | --- | --- | --- | --- |
| PET/AgNWs | 10.3 | 127.7 | 78.8 | 1.3E-006 | 2.2E-007 |
| PET/AgNWs/α-ZnO/Ag | 2.8 | 84.9 | 2108 | 2.0E-006 | 8.05E-008 |
